# Supplementary material for: Flexibility in task performance and division of labour in a cooperatively breeding cichlid
Source: Behav Ecol Sociobiol. 2026 Apr 9;80(4):49. doi: 10.1007/s00265-026-03723-7 (PMC13658308; doi:10.1007/s00265-026-03723-7)
Supplement: Supplementary file 1 [file 265_2026_3723_MOESM1_ESM.docx]

# Supplementary material

# Flexibility in task performance and division of labour in a cooperatively breeding cichlid

A. Ramesh^1*^ and B. Taborsky^1^

1. Behavioural Ecology, Institute of Ecology and Evolution, University of Bern, Wohlenstrasse 50A, CH-3032 Hinterkappelen, Switzerland

*Corresponding author. E-mail: aparajitha.ramesh@unibe.ch

Orcid

A. Ramesh: 0000-0002-7200-1366

B. Taborsky: 0000-0003-1690-8155

**Journal name: Behavioral Ecology and Sociobiology**

**b.**

**a.**

**Figure S1.** Hazard rates for specifying bouts for digging and defence events. The plots show the estimated hazard rates for behavioural persistence in (a) digging and (b) defence tasks. The hazard rate represents the conditional probability that individual transitions out of a given behaviour at time *t*, given that it has continued the behaviour up to that point. Both behaviours exhibit a steep decline in hazard rates within the first 10 seconds, after which the rates reach a relatively stable, lower baseline, which was used as a cut-off to determine bout length

**Figure S2.** Classification of bouts and following events, illustrated with two hypothetical individuals. Bouts are classified as clusters of the same behaviour (e.g. black or red) performed by the same size class or individual within 10 seconds (cf. hazard rates in Fig. S1). A switch from one behaviour to another (e.g. black to red), even within 10 seconds, marks the start of a new bout. ‘Following’ events occur when a different individual or size class performs a behaviour within 5 seconds after the initiator's action. Each bout includes an identified ‘**initiator’ and ‘follower’**, and these roles can vary across individuals or size classes within a single trial.

**Figure S3.** Results of the sensitivity analysis to assess the robustness of our operational definition of a "following event. We varyied the allowable time lag between an initiator's action and the next action of a second individual. Specifically, we tested temporal thresholds of 3, 10, 15, and 20 seconds (5 seconds is the default in the main text), within which a subsequent behaviour by another individual or size class was considered as a follow

**Figure S4.** a. An example of a group (52HH) showing consistency in digging by size class across the last two rounds (there were no digging events in the first round) and of another group (71SS) with a lack of consistency across size classes. b. An example in which division of labour occurred in a group (52HH). c. An example of group 62LL, in which there was division of labour in round 2 but not in rounds 1 & 3 – plotted over time to visualise spontaneous DOL, different colours represent different helper classes.

a)

b)

**Figure S5**. a&b) The two tasks performed over time across the rounds in different groups. This plot gives the overall high-resolution data collected within each trial for different groups. Each point represents an observation of task performed by an individual / size class given by the colour.
